# Promoting Evidence-Based Tobacco Cessation Treatment in Community Mental Health Clinics: Protocol for a Prepost Intervention Study

**DOI:** 10.2196/44787

**Published:** 2023-05-12

**Authors:** Faith Dickerson, Stacy Goldsholl, Christina T Yuan, Arlene Dalcin, Benjamin Eidman, Eva Minahan, Joseph V Gennusa 3rd, Elizabeth Mace, Bernadette Cullen, A Eden Evins, Corinne Cather, Nae-Yuh Wang, Emma M McGinty, Gail L Daumit

**Affiliations:** 1 Department of Psychology Sheppard Pratt Baltimore, MD United States; 2 Division of General Internal Medicine Johns Hopkins University School of Medicine Baltimore, MD United States; 3 Department of Health Policy and Management Johns Hopkins Bloomberg School of Public Health Baltimore, MD United States; 4 Department of Psychiatry and Behavioral Sciences Johns Hopkins University School of Medicine Baltimore, MD United States; 5 Department of Psychiatry Massachusetts General Hospital Boston, MA United States

**Keywords:** coaching, expert consultation, implementation, motivational interviewing, self-efficacy, serious mental illness, smoking cessation, system-level intervention, tobacco dependence

## Abstract

**Background:**

Tobacco smoking is highly prevalent among persons with serious mental illness (SMI) and is the largest contributor to premature mortality in this population. Evidence-based smoking cessation therapy with medications and behavioral counseling is effective for persons with SMI, but few receive this treatment. Mental health providers have extensive experience working with clients with SMI and frequent treatment contacts, making them well positioned to deliver smoking cessation treatment. However, few mental health providers feel adequately trained to deliver this treatment, and many providers believe that smokers with SMI are not interested in quitting or have concerns about the safety of smoking cessation pharmacotherapy, despite substantial evidence to the contrary.

**Objective:**

We present the protocol for the pilot “IMPACT” (Implementing Action for Tobacco Smoking Cessation Treatment) study, which aims to pilot test a multicomponent implementation intervention to increase the delivery of evidence-based tobacco smoking cessation treatment in community mental health clinics.

**Methods:**

We are using a prepost observational design to examine the effects of an implementation intervention designed to improve mental health providers’ delivery of the following four evidence-based practices related to smoking cessation treatment: (1) assessment of smoking status, (2) assessment of willingness to quit, (3) behavioral counseling, and (4) pharmacotherapy prescribing. To overcome key barriers related to providers’ knowledge and self-efficacy of smoking cessation treatment, the study will leverage implementation strategies including (1) real-time and web-based training for mental health providers about evidence-based smoking cessation treatment and motivational interviewing, including an avatar practice module; (2) a tobacco smoking treatment protocol; (3) expert consultation; (4) coaching; and (5) organizational strategy meetings. We will use surveys and in-depth interviews to assess the implementation intervention’s effects on providers’ knowledge and self-efficacy, the mechanisms of change targeted by the intervention, as well as providers’ perceptions of the acceptability, appropriateness, and feasibility of both the evidence-based practices and implementation strategies. We will use data on care delivery to assess providers’ implementation of evidence-based smoking cessation practices.

**Results:**

The IMPACT study is being conducted at 5 clinic sites. More than 50 providers have been enrolled, exceeding our recruitment target. The study is ongoing.

**Conclusions:**

In order for persons with SMI to realize the benefits of smoking cessation treatment, it is important for clinicians to implement evidence-based practices successfully. This pilot study will result in a set of training modules, implementation tools, and resources for clinicians working in community mental health clinics to address tobacco smoking with their clients. Trial Registration: ClinicalTrials.gov NCT04796961; https://clinicaltrials.gov/ct2/show/NCT04796961

**Trial Registration:**

ClinicalTrials.gov NCT04796961; https://clinicaltrials.gov/ct2/show/NCT04796961

**International Registered Report Identifier (IRRID):**

DERR1-10.2196/44787

## Introduction

Tobacco smoking is the largest contributor to premature mortality among persons with serious mental illness (SMI) [1-4]. An estimated 53% of US adults with SMI smoke tobacco [5], compared to 14% of US adults overall [6]. In general, US smoking rates have declined dramatically over the past 50 years, but smoking has persisted at consistently high rates among those with SMI [7,8], even though the majority of persons with SMI who smoke would like to quit [9]. Pharmacotherapy combined with behavioral counseling is effective and safe for people with SMI [10-13] and increases abstinence rates by up to 7 times over behavioral counseling alone [11,14].

The provision of effective, evidence-based smoking cessation treatment could substantially reduce the premature mortality experienced by people with SMI; however, few in this group receive evidence-based treatment [15-20]. In a large Maryland behavioral health system, less than 5% of persons with schizophrenia who smoke are prescribed evidence-based smoking cessation pharmacotherapy [8]. Limited available research shows similarly low rates of smoking cessation pharmacotherapy as well as behavioral smoking cessation counseling [21] delivery to the individuals with SMI who smoke in other states [15,18].

As many people with SMI receive the majority of their health care from the specialty mental health sector [22-24] and tobacco smoking is not typically a target behavior in mental health settings [21,22], it is critical to prepare mental health providers to deliver evidence-based smoking cessation treatment. Achieving this goal will require addressing knowledge and self-efficacy barriers among clinicians, including skepticism that people with SMI want to quit smoking, misperceptions about cessation treatment efficacy and safety for this group, and low self-efficacy to deliver evidence-based smoking cessation treatment for clients with SMI [15,25-28]. Mental health providers may also perceive smoking cessation treatment as outside their scope of practice [15,25-28]. Clinic-level barriers to treatment, such as lack of systems for assessing smoking status and interest in quitting among clients with SMI, may also need to be addressed [25,26]. Despite these known barriers, few published studies have evaluated implementation strategies to increase mental health providers’ delivery of evidence-based smoking cessation pharmacotherapy and counseling for people with SMI [29].

This protocol describes a pilot study of an implementation intervention of evidence-based smoking cessation treatment in community mental health clinics. We developed and are testing an implementation intervention designed to improve mental health providers’ knowledge, self-efficacy, and delivery of evidence-based smoking cessation treatment. The shorthand name for the study is “IMPACT” (Implementing Action for Tobacco Smoking Cessation Treatment).

## Methods

### Conceptual Framework

**Figure 1:**
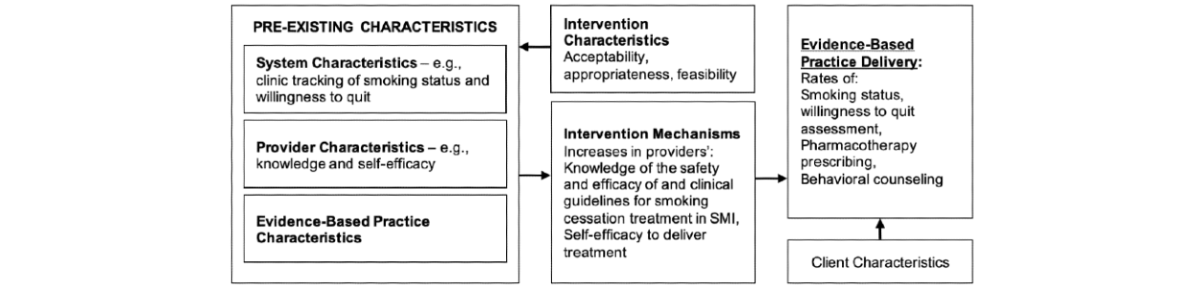
Gurses’ interdisciplinary conceptual framework of clinician’s compliance with evidence-based guidelines [30].

Our study is grounded in Gurses et al’s [30] model of interdisciplinary factors which promote clinicians’ compliance with evidence-based guidelines. As shown in Figure 1, the model posits the importance of preexisting characteristics of the system (eg, tools and technologies and physical environment), providers (eg, knowledge and attitudes), and guideline characteristics (eg, relative advantage and complexity). These factors affect the implementation intervention characteristics (ie, how and when a practice is implemented) and mechanisms that, in turn, affect the delivery of evidence-based practices.

### Pilot Study Design

This is a prepost study examining the effects of a 12-month implementation intervention on providers’ knowledge and self-efficacy and the mechanisms through which the implementation intervention is designed to improve the delivery of evidence-based smoking cessation treatment. We will also evaluate the effects of the implementation intervention on the delivery of evidence-based practices for smoking cessation. In addition, we will analyze measures of providers’ perceptions of the acceptability, feasibility, and appropriateness of the implementation strategies and the IMPACT evidence-based practices.

### Setting and Sample

The study is conducted in 5 community mental health clinics, all of which serve persons with SMI. Provider participants are employees of the community mental health organizations, including leaders and mental health clinicians (prescribers, including psychiatrists, nurse practitioners, and primary care physicians, therapists, and other clinicians, including licensed counselors, psychologists, social workers, and nurses). Data will also be analyzed from the clients served by the clinics around their receipt of the evidence-based practices. The research was approved by the Johns Hopkins University School of Medicine Institutional Review Board (IRB) (#IRB00231836).

### Evidence-Based Practices

The intervention includes the following four evidence-based practices for smoking cessation that are based on consensus guidelines, recommendations for persons with SMI, and recent clinical trials [10,11,13,31-33]:

1. Assessment of smoking status involves asking adult clinic clients if they currently smoke tobacco and, if they respond yes, further assessing the severity of tobacco dependence [34].
2. Assessment of willingness to quit uses stage-of-change questions [34]. In this project, we classify those endorsing setting a quit date within 12 weeks as ready for active cessation treatment [35]. We targeted willingness to quit within 12 weeks rather than the standard 30 days in order to include motivational enhancement strategies for those not ready to commit to a quit date within 1 month and to give adequate time to complete behavioral counseling and achieve smoking cessation for those interested in quitting [36].
3. The focus of the behavioral counseling will vary depending on clients’ willingness to quit. For clients who are not interested in quitting within 12 weeks, behavioral counseling entails an immediate, brief session (<10 minutes) using motivational interviewing techniques to explore the pros and cons of smoking, elicit client concerns about their smoking, and identify potential benefits of quitting [37]. The provider will also discuss the resources available to help the client if they decide to work toward quitting, including the benefits of using smoking cessation medications. Those clients who express a willingness to quit will be referred for ongoing smoking cessation behavioral counseling [36] and smoking cessation pharmacotherapy. The counseling sessions, which can occur remotely or in person, are cognitively tailored for those with SMI and address standard topics (eg, identifying personal reasons to quit, addressing smoking triggers, coping with nicotine withdrawal, and preparing for the quit date) [38]. Clients who do not quit are encouraged to continue counseling if they are interested in making a future quit attempt. When a client quits, counseling then focuses on relapse prevention, including refusal skills, responding to permission-giving beliefs, and developing personalized relapse prevention plans.
4. The fourth evidence-based practice is pharmacotherapy prescribing, in which a prescribing physician or nurse practitioner meets with clients undergoing smoking cessation behavioral counseling to encourage use of 1 of the following 3 Food and Drug Administration (FDA)–approved treatments: varenicline with or without nicotine replacement therapy (NRT), bupropion SR with or without NRT, or NRT alone [34]. When NRT is used, combination NRT (transdermal patch + lozenge or gum) is recommended. Medication choice is made by the prescriber in a collaborative discussion with the client, taking into account factors such as differential effectiveness, potential side effects, and clinical profile (eg, seizure history). Therapists and prescribers regularly share care of clients and will communicate about smoking cessation plans. Medication may continue for 1 year or longer as relapse rates are high without ongoing pharmacotherapy, particularly for those with SMI [10]. Those not willing to quit within 12 weeks will be offered, and if interested, referred for, a 1-month trial of varenicline to increase their readiness to quit [39]. The goal is that all clients who smoke should be offered treatment with a first-line smoking cessation pharmacotherapy, preferentially varenicline or combination NRT [40].

### Implementation Strategies

#### Overview

To improve mental health providers’ knowledge, self-efficacy, and delivery of evidence-based practices, the study will employ the following five implementation strategies: (1) training on evidence-based smoking cessation treatment, including an innovative web-based avatar module for motivational interviewing practice; (2) a tobacco smoking treatment protocol; (3) expert consultation for prescribers and therapists; (4) coaching; and (5) organizational strategy meetings (OSMs). A more detailed description of each component is outlined below.

#### Training

Trainings are conducted by study staff. All provider participants in the IMPACT project complete 1 hour of prerecorded web-based training modules. Then, therapists and prescribers use a video conferencing platform to participate in real-time training on delivering smoking cessation behavioral counseling, prescribing smoking cessation pharmacotherapy, and using motivational interviewing counseling skills when having conversations with clients about their smoking. Trainings are housed in a web-based management system that enables tracking of provider participation as well as continuing education credit. The training outline is shown in Textbox 1.

The real-time motivational interviewing training is supplemented with a web-based avatar practice module developed in partnership with Kognito [41,42]. The module includes a 15-minute didactic component during which clinicians are reminded about motivational interviewing techniques that can be used to guide clients with SMI toward health behavior changes and a 15-minute conversation simulation during which providers assume the role of a provider avatar and use motivational interviewing to discuss smoking cessation with a client avatar. A web-based avatar with simulated conversations enables the learner to use and receive immediate feedback about how different responses in a clinical encounter affect a conversation with a patient in which smoking cessation is discussed. The avatar is appealing and can be accessed at the convenience of the learner. Clinicians are encouraged to use the platform once per month throughout the intervention.

**Outline of provider training.**

**Prescribers (psychiatrists and nurse practitioners):**

- Online training modules (1 hour)
  Module 1: Why IMPACT (Implementing Action for Tobacco Smoking Cessation Treatment)?
  Module 2: Evidence-based treatment for smoking cessation for persons with serious mental illness (SMI)
  Module 3: Assessing smoking status willingness to quit
  Module 4: Pharmacotherapy for smoking cessation
- Real-time trainings (in person or videoconferencing platform) (2 hours)
  Training 1 (1 hour): Facilitating smoking cessation conversations
  Training 2 (1 hour): Motivational interviewing approach to smoking cessation pharmacotherapy
- Online motivational interviewing avatar (recommended monthly 15-minute practice sessions)

**Therapists (psychologists, social workers, counselors):**

- Online training modules (1 hour)
  Module 1: Why IMPACT?
  Module 2: Evidence-based treatment for smoking cessation for persons with SMI
  Module 3: Assessing smoking status willingness to quit
  Module 4: Pharmacotherapy for smoking cessation
- Real-time trainings (in person or zoom) (4 hours)
  Training 1 (2 hours): Facilitating smoking cessation conversations and an overview of behavioral counseling
  Training 2 (2 hours): Motivational interviewing approach to smoking cessation behavioral counseling
- Online motivational interviewing avatar (recommended monthly 15-minute practice sessions)

#### Tobacco Smoking Treatment Protocol

The research team developed a tobacco smoking treatment protocol to assist clinicians in delivering evidence-based smoking cessation treatment. This protocol includes a resource guide that provides an overview of the IMPACT program and will help clinicians navigate their work on the project. Clients who report being current smokers (ie, have smoked a tobacco product in the past 7 days) are assessed for their willingness to quit. Based on client responses to the tobacco smoking status and willingness-to-quit assessment, instructions help clinicians facilitate brief smoking cessation conversations; all clients interested in quitting will be offered treatment. Clinicians are provided manuals for delivering smoking cessation behavioral counseling sessions and prescribing pharmacotherapy.

The smoking cessation behavioral counseling manual includes the outline for 13 session topics that can be delivered flexibly based on the client’s current smoking status and the provider’s judgment about what would be most helpful (see Table 1). A smoking cessation session may be “standalone” or part of a regularly scheduled therapy session and is designed to be 10-15 minutes in duration. Each session is expected to include an assessment of the client’s past week’s average cigarettes smoked per day, current smoking status and review of quit attempts since the previous visit, new session content, and collaborative identification of a smoking-related behavioral goal to focus on until the next therapy session.

**Table 1:** Topics of behavioral counseling sessions.

Session	Topic^a^
1	Introduction: positives and negatives of smoking
2	Health effects of smoking and benefits of quitting
3	Smoking cessation pharmacotherapy to help you quit
4	Barriers to quitting and the 4 D’s
5	Identifying triggers and high-risk situations
6	Smoking cessation medication adherence and coping with side effects
7	Planning for the quit day
8	Withdrawal symptoms and coping with withdrawal symptoms
9	Quit day follow-up
10	Developing a “Stay Quit” plan
11	Permission-giving beliefs and how to respond to them without smoking
12	Dealing with slips
13	Coping with symptoms of SMI^b^ and staying strong

^a^Order and selection of topics to be made by therapists based on their clinical judgment and on the smoking status of the client.

^b^SMI: serious mental illness.

#### Expert Consultation

Expert consultation is available for prescribers and therapists to discuss issues and ask questions related to smoking cessation pharmacotherapy and behavioral counseling. Study team members who are experts in smoking cessation pharmacotherapy and behavioral counseling are available during the 12-month intervention to conduct as-needed phone or email consultation with clinicians.

#### Coaching

Coaching is available for therapists and prescribers to discuss specific client cases, develop smoking cessation behavioral counseling skills, and answer questions about prescribing smoking cessation pharmacotherapy. The coaching sessions, which will be offered monthly through a videoconferencing platform, will focus on skill development, overcoming barriers to providing evidence-based smoking cessation treatment, and additional training as requested. To increase participation, we aim to incorporate coaching sessions into regularly scheduled clinic meetings but will schedule individual coaching sessions as needed. The expectation is that providers attend at least one session per quarter.

#### Framework of OSMs

OSMs are designed to improve engagement in practice change. These meetings will occur monthly and consist of a 2-to-3–member clinic leadership team, including providers and staff leaders. OSMs will be a chance for the study team to work with the clinic to identify successful organizational-level processes, share implementation data, provide feedback, and problem-solve to overcome identified barriers to program delivery.

### Data Collection

Data will be obtained from several sources, including provider surveys, provider interviews, web-based data collection regarding provider participation in training, and client data from the electronic medical record. Providers will be surveyed either by email with instructions and a link to the study assessments in the REDCap software or by paper-and-pencil, depending on-site preference; these assessments will be administered at baseline and at 12 months. Table 2 presents a summary of data collection and measures. Assessments will include measures of providers’ knowledge about smoking cessation, self-efficacy about delivering smoking cessation treatment, motivation to deliver evidence-based practices, and beliefs about and confidence in using motivational interviewing. The surveys will also assess providers’ views of the acceptability, appropriateness, and feasibility of the intervention and of the implementation strategies, implementation climate of the organization, and providers’ social network ties within the organization. A convenience sample of providers and clinic leaders also will be interviewed at baseline and 12 months to elicit their perceptions of the intervention, implementation barriers and facilitators, and the implementation strategies used to overcome barriers.

**Table 2:** Study data collection.

Measure	Description	Participants	Timing
Provider interviews	Semistructured interviews using a standard protocol that will elicit providers’ perceptions of barriers to smoking cessation treatment and implementation strategies to overcome barriers.	Selected providers, clinic director	Baseline, 12 months
Demographic characteristics	Age, gender, race or ethnicity, length of time at program, role in program, years at clinic, work hours, previous motivational interviewing training	All providers, clinic leaders	Baseline
Knowledge of evidence-based smoking cessation treatment	A 16-item scale developed by our team to assess knowledge	All providers	Baseline, 12 months
Self-efficacy	To deliver evidence-based smoking cessation treatment, adapted version of Compeau and Higgins’ task-focused self-efficacy scale [43]	All providers	Baseline, 12 months
Implementation climate	A measure of the degree to which an organization supports evidence-based practice implementation [44]	All providers, selected clinic leaders	Baseline
Acceptability, appropriateness, and feasibility of the intervention implementation strategies	Measured with a brief 4-item practice instrument (AIM^a^, IAM^b^, FIM^c^) [45]	All providers, selected clinic leaders	Baseline, 12 months
Acceptability, appropriateness, and feasibility of the evidence-based practice	Measured with a brief 4-item practice instrument (AIM, IAM, FIM) [45]	All providers, selected clinic leaders	Baseline, 12 months
Clinic social network	A 4-question survey used to collect data about social networks	All providers, selected clinic leaders	Baseline, 6, 12 months
Motivation measures	Assessment of degree of agreement or disagreement with statements that deal with aspects of the intervention to improve use of evidence-based smoking cessation treatment	All providers	Baseline, 12 months
Beliefs about the Motivational Interviewing Questionnaire	A 7-question survey assessing the extent to which each person agrees with statements about motivational interviewing.	All providers	Baseline, 12 months
Importance and confidence of using motivational interviewing	A 6-question survey assessing the importance and confidence each person has to deliver motivational interviewing.	All providers	Baseline, 3, 6, 12 months
Avatar motivational interviewing performance measurements	From use of motivational interviewing techniques in simulated web-based conversations	All providers	Baseline, 12 months
Motivational Interviewing Treatment Integrity Tool	Validated tool used to measure fidelity to motivational interviewing from Standardized Actor Interviews	All providers	Baseline, 12 months
Fidelity to behavioral smoking cessation counseling	Standardized Actor Interviews will be conducted to assess fidelity to smoking cessation behavioral counseling sessions	All providers	Baseline, 12 months
Guideline-concordant smoking cessation treatment	Smoking status assessment rates Willingness-to-quit assessment rates Prescription of smoking cessation medication rates Behavioral counseling rates	All clinic clients	Baseline through 12 months

^a^AIM: Acceptability of Intervention Measure.

^b^IAM: Intervention Appropriateness Measure.

^c^FIM: Feasibility of Intervention Measure.

Data on providers’ competency using motivational interviewing skills will be obtained from scheduled avatar-simulated conversations and from standardized actor phone interviews, which will occur at baseline and 12 months at a minimum. Standardized client scenarios will be provided to actors that reflect the kind of situations community mental health clinicians would likely encounter with clients who smoke; this method has demonstrated predictive validity in terms of providers’ performance with actual clients [46]. The research team will access data from the electronic medical record (EMR) system or site-specific documentation procedures to assess providers’ uptake of the evidence-based practices (eg, percentage of clinic visits where a smoking assessment is documented) and client-level data (eg, demographics and primary mental health diagnosis).

### Measures

The *primary outcomes* are changes in providers’ knowledge about and self-efficacy to deliver evidence-based smoking cessation treatment from baseline to 12 months.

*Secondary outcomes* include change in the delivery of the recommended four evidence-based practices: (1) assessment of smoking status for all adult clinic clients (with quarterly follow-up assessments for smokers not yet willing to quit), (2) assessment of willingness to quit for smokers, (3) receipt of behavioral counseling for those willing to quit within 12 weeks, and (4) receipt of pharmacotherapy for those willing to quit within 12 weeks and referral for pharmacotherapy for those not willing to quit in this time frame. These outcomes will be measured by clinic documentation during client visits (in the EMR or site-specific documentation) and assessed at baseline and throughout the implementation period. When available, baseline documentation will include data from 3-6 months prior to project implementation. Additional secondary measures are changes in the acceptability, appropriateness, and feasibility of the intervention implementation strategies and of the evidence-based practices as assessed through the survey instrument.

To assess motivational interviewing skills, we will use the Motivational Interviewing Treatment Integrity coding system [47]. We will code random 20-minute segments of audio-recorded interactions with standardized client actors. The same interviews will be used to assess fidelity to the components of an IMPACT evidence-based smoking cessation counseling session.

Perceived barriers and facilitators on the part of clinic providers and leaders will be assessed based on qualitative interviews with individual providers before the start of the implementation intervention and at 12 months, and also with information obtained in monthly OSMs. We will also collect data about providers’ and clients as shown in Table 2 including age, gender, race or ethnicity, and for providers, years worked at the clinic, and for clients, primary mental health diagnosis.

### Data Analysis

We will conduct 2 main analyses. First, we will use survey data to assess the effects of the implementation intervention (baseline and 12 months) on providers’ knowledge and self-efficacy, the mechanisms through which the implementation intervention is designed to improve the delivery of evidence-based smoking cessation treatment, using a generalized linear mixed effects modeling approach. The model will include a binary variable representing the prepost time points, fixed effects for the 5 study sites, and provider demographic characteristics. Second, we will evaluate the effects of the intervention (baseline and 12 months) on the implementation of evidence-based practices using a multilevel modeling approach. We will also assess the potential moderating effects of the implementation climate by adding appropriate interaction terms to the main models. We will use descriptive statistics to characterize staff perceptions of the acceptability, feasibility, and appropriateness of the implementation intervention strategies and the IMPACT evidence-based practices. Interview transcripts will be analyzed in MAXQDA (VERBI GmbH), using inductive coding to identify key themes. Survey analysis will be done using Stata 14 (StataCorp LLC) or SAS software (SAS Institute).

### Ethical Considerations

The study was approved by the Johns Hopkins University School of Medicine IRB on January 14, 2021 (IRB number 00231836). Community mental health leaders and clinical staff will be appropriately recruited and informed of the study by the study team, with a waiver of documentation of informed consent. Patient data will be obtained per an approved waiver of consent. These waivers were approved by the IRB.

Each participant will be assigned a unique study ID number for data collection. Interview data will be audio recorded and transcribed by an approved vendor. Names will be removed from transcripts. Standardized actor interview will be stored on a secure network and participants identified by study number only. Study data will not be presented in such a way that identity can be inferred. Mental health leader and staff study participants will be paid US $50 for completing each interview and US $25 for each standardized actor interview as applicable. They will be paid up to a maximum total of US $60 for completing surveys at 4 time points.

## Results

The study is underway at 5 clinic sites. More than 50 providers have been enrolled, exceeding our recruitment target. The study is ongoing. We anticipate that results will be reported in 2023 or 2024.

## Discussion

### Overview

Tobacco smoking is the leading cause of preventable mortality among persons with SMI and contributes to the reduced lifespan up to 25 years for persons in this population [1]. Evidence-based treatments for smoking cessation are available, safe, and efficacious for persons with SMI; however, few persons with SMI receive these treatments [38].

In order to realize the intended benefits of smoking cessation treatment for persons with SMI, it is important that mental health programs implement evidence-based practices. Current barriers faced by clinicians include not having the knowledge or confidence to deliver smoking cessation treatments, as well as misconceptions about the safety of smoking cessation medications and client interest in quitting [25-28]. There are also barriers at the organizational level; community mental health centers may not have standard systems for screening, monitoring, or treating tobacco smoking. In addition, the complexity of the evidence-based treatment, that is, the combination of behavioral counseling and pharmacotherapy, often delivered by different clinicians, contributes to the challenges of implementation [48]. In this pilot study, we will strive to address these barriers through the use of implementation strategies, including training about evidence-based smoking cessation treatment and motivational interviewing, a tobacco smoking treatment protocol, expert consultation, coaching, and OSMs.

### Strengths and Limitations

While evidence-based smoking cessation treatments are not provided routinely in mental health clinics, where people with SMI receive the majority of their health care, these settings provide opportunities for the delivery of these recommended practices. We have specifically designed the training materials for providers in the community mental health center setting and have tailored the counseling manual for delivery to persons with SMI. We also worked with the sites’ leadership to embed smoking cessation processes of care into the EMR and current workflow to increase clinician uptake. Using data from the EMR can provide an objective measure of smoking cessation evidence-based practices that are being implemented, enabling us to highlight areas of success and problem-solve barriers. At the completion of this study, we will have training modules, implementation tools, and resources for the delivery of smoking cessation treatment in community mental health centers. In order to address barriers to the implementation of these evidence-based practices, we are using a range of strategies, including asynchronous trainings and novel avatar simulations, to improve motivational interviewing counseling skills. We will collect data about the acceptability, appropriateness, and feasibility of both the evidence-based practices and implementation strategies that will inform future implementation efforts and planning for a randomized controlled trial.

In terms of limitations, our implementation intervention is effortful and multifaceted and may require additional supports to be carried out at other community mental health centers. The COVID-19 pandemic has created further challenges. As a result of pandemic-related restrictions, the study team had to adapt the implementation strategies to be delivered remotely rather than in-person, which, while being efficient to deliver, can make it more challenging to engage clinicians. Clinicians also had to adapt the counseling sessions to be delivered remotely if needed, which can be an added barrier for those less comfortable with technology. We also recognize that it may be difficult for providers to prioritize smoking cessation given other time-sensitive client concerns, such as those related to acute psychiatric symptoms and mental health crises [49]. Clinician challenges in delivering smoking cessation treatment may be exacerbated by staff shortages, which have occurred in many community mental health clinics during the pandemic and which have led to increased caseloads and other agency-related pressures. More generally, there are difficulties changing the culture in mental health organizations, many of which have been permissive about tobacco smoking in the past, in order to facilitate more active promotion and delivery of smoking cessation treatments [15]. Finally, our prepost design does not allow us to make causal inferences about the effects of our implementation strategies on outcomes.

### Conclusion

The IMPACT study will provide evidence about the use of implementation strategies to increase providers’ knowledge, self-efficacy, and delivery of the evidence-based practices for smoking cessation treatment for persons with SMI in community mental health center settings.

## Abbreviations

EMR: electronic medical record
IMPACT: Implementing Action for Tobacco Smoking Cessation Treatment
IRB: institutional review board
NRT: nicotine replacement therapy
OSM: organizational strategy meeting
SMI: serious mental illness

## Data Availability

The data sets generated and analyzed during this study will be available from the corresponding author on reasonable request with appropriate institutional review board approval and data use agreement.
